# OSCARinED: a policy and practice review of an emergency department-based critical care framework for critically ill non-trauma patients

**DOI:** 10.3389/fmed.2026.1872523

**Published:** 2026-07-09

**Authors:** Johann J. Kemper, Mark Michael, Ines Elsemann, Leon Ghobadi, Philipp Kümpers, Michael Reindl, Bernhard Kumle, Martin Pin, Michael Bernhard

**Affiliations:** 1 Emergency Department, University Hospital of Duesseldorf, Heinrich-Heine University, Duesseldorf, Germany; 2 Department of Medicine D, Division of General Internal and Emergency Medicine, University Hospital Muenster, Muenster, Germany; 3 Department of Emergency and Acute Medicine, AMEOS Klinikum St Josef Oberhausen, Oberhausen, Germany; 4 Department of Emergency and Acute Medicine, Hospital of Villingen-Schwenningen, Villingen-Schwenningen, Germany; 5 Department of Emergency and Acute Medicine, Florence-Nightingale Hospital, Duesseldorf, Germany

**Keywords:** emergency medicine, critical care, emergency department boarding, emergency critical care, implementation framework, bedside cognitive aid, handover, patient safety

## Abstract

Critically ill non-trauma patients are increasingly managed in the emergency department (ED) for prolonged periods because of hospital crowding, intensive care unit (ICU) capacity constraints, and diagnostic or procedural delays. During this interval, ED teams are required to provide emergency critical care (ECC) that extends beyond initial resuscitation and includes continued organ support, target-based reassessment, prevention of secondary complications, and structured handover. However, unlike trauma and cardiac arrest care, ongoing ECC for boarded non-trauma patients is less consistently supported by ED-specific practice frameworks. This Policy and Practice Review introduces OSCARinED, a pragmatic framework designed to organize ED-based ECC after initial ABCDE stabilization. OSCARinED integrates seven recurrent care domains: oxygenation and ventilation; sedation and analgesia; catecholamines, fluid therapy, and catheter management; anti-infective therapy; risk-adapted positioning and prophylaxis; electrolyte, blood gas, glucose, and nutrition management; and delirium prevention and treatment. The framework links trigger recognition and framework activation to explicit treatment targets, scheduled reassessment, documentation, and disposition planning. OSCARinED is intended as a safety bridge during ED boarding, not as a substitute for ICU admission. Its implementation may support standardization of care, reduce omission-prone tasks, improve handover reliability, and provide a structure for local quality improvement. Future studies should evaluate OSCARinED using implementation-science designs, focusing on process reliability, team performance, patient-centered outcomes, and ED operational endpoints.

## Highlights

- Prolonged boarding of critically ill non-trauma patients creates a practice gap between initial ED resuscitation and ICU-level continuity of care.
- OSCARinED provides an ED-specific structure for emergency critical care after initial ABCDE stabilization.
- The framework links seven care domains to explicit treatment targets, reassessment intervals, handover, and disposition planning.
- OSCARinED should be evaluated as an implementation and patient-safety intervention rather than as a replacement for ICU admission.

## Introduction

Prolonged boarding of critically ill patients in the emergency department (ED) has become a recurrent systems-level challenge across healthcare settings. Although patients with ongoing organ dysfunction should ideally be transferred rapidly to an intensive care unit (ICU) or another high-acuity care area, limited ICU capacity, organizational constraints, staffing shortages, seasonal demand, and diagnostic or procedural delays frequently result in extended ED stays. During this interval, ED teams must deliver emergency critical care (ECC) that extends beyond initial resuscitation and increasingly resembles early ICU-level management.

Delayed ICU transfer has been associated with adverse outcomes, including increased ICU and hospital mortality. Systematic reviews report ED boarding times ranging from several hours to more than 8 h among critically ill patients awaiting ICU admission (1, 2). These delays create a vulnerable transition period in which advanced monitoring, ventilatory support, vasoactive therapy, infection management, prophylaxis, metabolic control, delirium prevention, and repeated handovers must be organized reliably within the operational constraints of the ED.

Consequently, the ED is increasingly functioning as a site of early critical care delivery. However, unlike trauma care, cardiac arrest care, or sepsis pathways, the ongoing management of critically ill non-trauma patients after initial ABCDE stabilization is less consistently structured. This creates a practical and policy-relevant gap: EDs require feasible frameworks that translate critical care priorities into target-based workflows, documentation prompts, reassessment intervals, and handover structures that can be applied even when dedicated ED-based critical care units are unavailable. This gap is particularly relevant because ED boarding exposes critically ill patients to a prolonged phase in which organ support, antimicrobial therapy, prophylaxis, metabolic control, delirium prevention, repeated reassessment, and interprofessional handover must be maintained under time pressure, interruptions, and competing operational priorities. Delayed ICU transfer and prolonged ED boarding have been associated with adverse outcomes, but the practical mechanism by which ED teams can reliably maintain recurrent critical care tasks after initial ABCDE stabilization remains less clearly operationalized. A domain-based framework may therefore help make these recurrent tasks explicit, reduce omission-prone care processes, and support coherent disposition planning until ICU admission, transfer to another high-acuity area, clinical stabilization, or alternative disposition.

### Emergency critical care as a response to ED boarding

Emergency Critical Care (ECC) has emerged as a response to this gap. ECC can be understood as the provision of time-sensitive critical care interventions in the ED during the early phase of critical illness and while awaiting ICU admission, transfer to another high-acuity unit, stabilization, or alternative disposition. It includes airway and ventilatory management, vasoactive therapy, invasive or non-invasive hemodynamic monitoring, infection management, organ-supporting therapies, and repeated goal-directed reassessment.

Several institutions have implemented dedicated ED-based critical care programs or ED-ICUs, with encouraging effects on patient outcomes and resource utilization (3–9). These models represent structural solutions that typically rely on dedicated space, specialized staffing, enhanced monitoring capacity, defined governance, and integration with hospital-wide critical care pathways. They demonstrate that critically ill ED patients may benefit from early critical care expertise before conventional ICU admission. However, ED-ICU models, critical care resuscitation units, and specialized ECC programs are resource-intensive and not universally available, particularly in EDs where critically ill patients must be managed within existing resuscitation rooms, observation areas, or high-dependency spaces. OSCARinED addresses this remaining implementation gap. It is not a location-based ED-ICU model, but a process-oriented practice framework and bedside cognitive aid intended to structure ongoing ECC within existing ED workflows. In contrast to disease-specific pathways for trauma, cardiac arrest, or sepsis, OSCARinED focuses on recurrent cross-cutting care domains after initial ABCDE stabilization and links these domains to documented targets, scheduled reassessment, handover, and disposition planning. This rationale supports the need for pragmatic, scalable practice frameworks that help ED teams deliver structured ECC without requiring the immediate creation of dedicated ED-ICU infrastructure. Such frameworks should define when to activate ECC, which domains require repeated review, how targets should be documented, and how responsibility should be transferred during handover and disposition planning. Table 1 summarizes how OSCARinED relates to existing ED-based critical care models, ED-ICU concepts, ICU-derived safety mnemonics, and disease-specific emergency pathways. The comparison highlights that existing ED-ICU and emergency critical care models primarily address the problem through dedicated structures, staffing, and governance, whereas ICU-derived mnemonics and disease-specific pathways address selected safety or syndrome-specific tasks. OSCARinED addresses the remaining process gap by providing a diagnosis-agnostic, ED-specific structure for recurrent target-setting, reassessment, documentation, handover, and disposition planning within existing ED workflows, particularly when ICU transfer is delayed or when safe non-ICU disposition is being considered. These approaches are complementary rather than mutually exclusive: structural ED-ICU models demonstrate the value of early critical care delivery in the ED, whereas OSCARinED addresses the remaining process gap by providing a diagnosis-agnostic framework for recurrent target-setting, reassessment, documentation, handover, and disposition planning after initial ABCDE stabilization. Against this background, OSCARinED should be understood as a complementary process framework rather than as a competing structural model. Its purpose is to make recurrent critical care tasks explicit, transferable, and reassessable during the vulnerable interval between initial ED stabilization and definitive disposition.

**Table 1:** Comparison of ED-based critical care models and OSCARinED.

Model/framework	Setting	Target population	Infrastructure/staffing	Studied outcomes or purpose	Limitations
ED-ICU/ED-based intensive care unit	Dedicated ICU-like area within or adjacent to the ED	Critically ill ED patients requiring early critical care	Dedicated beds, advanced monitoring, specialized medical and nursing staff, and defined governance	Improved survival, reduced ICU utilization, shorter hospital or ICU length of stay, and improved resource use have been reported in selected settings	Resource-intensive; requires dedicated infrastructure and staffing; not universally feasible
Critical care resuscitation unit	Specialized resuscitation or critical care unit focused on early stabilization and disposition coordination	Highly unstable patients requiring advanced resuscitation, organ support, and rapid disposition planning	Specialized team, advanced monitoring, procedural capability, and transfer coordination	Stabilization, safe transfer, disposition coordination, and sustainability of care delivery	Requires dedicated structure, staffing, and institutional governance
Emergency critical care nursing model	ED-based care model emphasizing critical care nursing expertise during boarding	Critically ill patients remaining in the ED after initial stabilization	Critical care-trained ED nurses, staffing models, protocols, and interprofessional escalation pathways	Improved care continuity and potential outcome benefits during ED boarding	Depends on local staffing, training, and nursing resources
ICU-derived FAST HUG/FAST HUG EACH HOUR	ICU bedside mnemonic and safety review	Critically ill ICU patients	ICU environment, ICU staff, and established critical care routines	Daily safety review and prevention of omission-prone ICU tasks	Not ED-specific; assumes ICU infrastructure, staffing, and workflow
Disease-specific ED pathways	ED trauma, cardiac arrest, sepsis, stroke, or other syndrome-specific pathways	Patients with defined diagnoses or time-critical syndromes	Protocol-dependent; often embedded into disease-specific team activation and quality metrics	Guideline adherence, time to intervention, and disease-specific outcomes	Do not comprehensively structure cross-cutting ongoing critical care after ABCDE stabilization
OSCARinED	Existing ED resuscitation room, observation area, high-dependency space, or boarding workflow	Critically ill non-trauma ED patients after initial ABCDE stabilization	No dedicated ED-ICU required; requires monitoring capacity, role allocation, documentation, reassessment cadence, and interprofessional training	Intended to improve process reliability, target documentation, reassessment, handover quality, disposition planning, and patient safety; prospective validation required	Not yet validated as an outcome-improving intervention

This conceptual comparison summarizes how OSCARinED relates to selected ED-based critical care structures, ICU-derived safety concepts, and disease-specific emergency pathways. The table is intended to clarify the remaining process gap addressed by OSCARinED and does not represent a systematic evidence grading. ABCDE, airway, breathing, circulation, disability, exposure; ECC, emergency critical care; ED, emergency department; ED-ICU, emergency department-based intensive care unit; FAST HUG, feeding, analgesia, sedation, thromboembolic prophylaxis, head-of-bed elevation, ulcer prophylaxis, glycemic control; ICU, intensive care unit; OSCARinED, oxygenation and ventilation, sedation and analgesia, catecholamines, fluid therapy and catheter management, anti-infective therapy, risk-adapted positioning and prophylaxis, electrolyte, blood gas, glucose and nutrition management, delirium prevention and management.

### Development and scope of the OSCARinED framework

**Figure 1:**
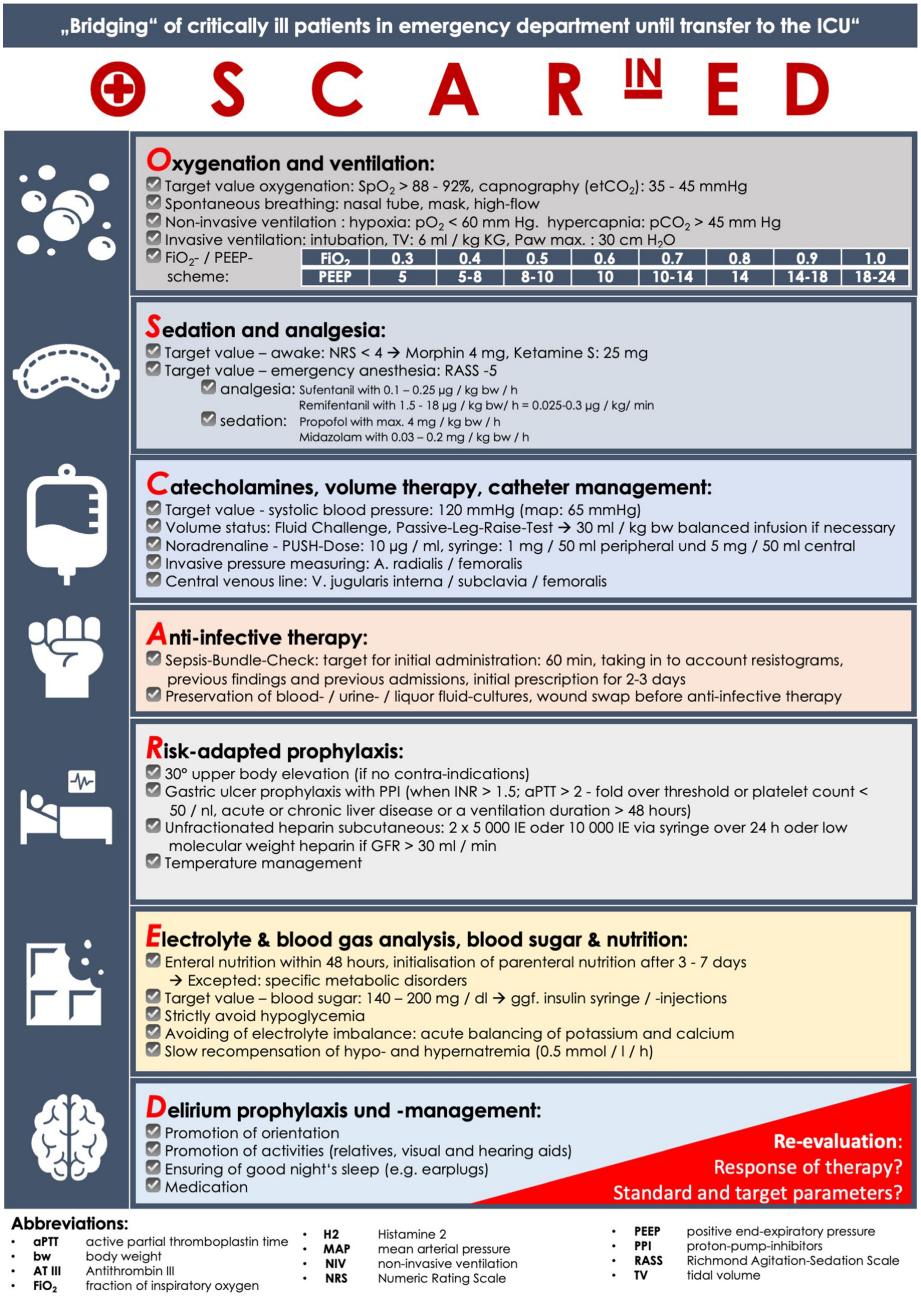
OSCARinED policy and practice framework for emergency critical care (ECC) in the emergency department (ED). OSCARinED is a bedside cognitive aid and practice framework designed to structure ECC for critically ill non-trauma patients who remain in the ED after initial stabilization and before ICU transfer, clinical stabilization, transfer to another high-acuity unit, or alternative disposition. The acronym summarizes key domains for repeated assessment and goal-directed management: O, oxygenation and ventilation; S, sedation and analgesia; C, catecholamines, fluid therapy, and catheter management; A, anti-infective therapy; R, risk-adapted prophylaxis and positioning; E, electrolyte and blood gas analysis, blood glucose control, and nutrition; and D, delirium prophylaxis and management. The bedside checklist implementing the OSCARinED framework is intended to support target definition, documentation, structured reassessment, and handover during prolonged ED stays. Re-evaluation should be performed at predefined intervals, after clinical deterioration, and after major changes in organ support, procedures, transport, handover, or disposition planning (aPTT, activated partial thromboplastin time; AT III, antithrombin III; bw, body weight; ECC, emergency critical care; ED, emergency department; FiO₂, fraction of inspired oxygen; GFR, glomerular filtration rate; ICU, intensive care unit; MAP, mean arterial pressure; NIV, non-invasive ventilation; NRS, Numeric Rating Scale; PEEP, positive end-expiratory pressure; PPI, proton pump inhibitor; RASS, Richmond Agitation-Sedation Scale; TV, tidal volume).

This Policy and Practice Review introduces OSCARinED as a pragmatic, evidence-informed practice framework and bedside cognitive aid for structuring ECC in critically ill non-trauma patients during prolonged ED stays. Building on the ICU-derived FAST-HUG framework (10) and its expansion to “FAST HUG EACH HOUR” (11), OSCARinED was developed as a lean, ED-focused mnemonic to structure post-resuscitation room care (Figure 1): (O)xygenation and ventilation, (S)edation and analgesia, (C)atecholamines, vascular access, catheter management and fluids, (A)nti-infective therapy, (R)isk-adapted positioning and prophylaxis, (E)lectrolyte and metabolic management, and (D)elirium prevention and management. The OSCARinED acronym and its practical bedside use were first introduced in a German-language review on ECC and bridging of critically ill ED patients to ICU admission (12). The present Policy and Practice Review expands this earlier concept into an international, ED-focused framework by contextualizing the acronym within ED boarding, implementation requirements, reassessment cycles, disposition planning, and quality indicators.

### Literature-gathering approach and framework development

OSCARinED was developed through a narrative, practice-oriented synthesis of relevant literature rather than through a systematic review. The author group purposively assembled contemporary guidelines, systematic reviews, landmark clinical studies, ICU-derived safety concepts, ED boarding literature, studies on ED-ICU and ECC models, and practice-oriented publications relevant to the individual OSCARinED domains. Literature was selected based on clinical relevance, applicability to ED workflows, recency where appropriate, and relevance to time-sensitive or omission-prone tasks during prolonged ED stays. Because this was not a systematic review, no predefined database search strategy, search date range, formal search-string protocol, or formal inclusion/exclusion algorithm was applied. Institutional protocols and local practice experience informed feasibility considerations but were not treated as independent evidence.

This approach was intended to translate established critical care principles into a pragmatic ED-focused framework after initial ABCDE stabilization, not to provide a comprehensive evidence grading. During this narrative process, the authors did not identify a prospectively validated ED-specific post-resuscitation framework for ongoing ECC in critically ill non-trauma patients that was directly comparable to OSCARinED.

### Conceptual definition and intended use

OSCARinED was therefore conceptualized as a process-oriented practice framework and bedside cognitive aid rather than as a clinical guideline, validated care bundle, or ED-ICU structural model. Accordingly, OSCARinED should be understood as an organizing framework for repeated target-setting, reassessment, documentation, and handover. The accompanying checklist is an implementation tool for bedside use, whereas the framework itself does not prescribe disease-specific treatment algorithms or replace clinical judgment, local standard operating procedures, or ICU admission pathways.

The resulting OSCARinED domains are not intended to replace disease-specific guidelines or ICU admission pathways. Rather, they provide a structured bridge between initial resuscitation and definitive disposition, with repeated reassessment as the central safety mechanism. Where clinically appropriate, this structured reassessment may also support de-escalation and safe non-ICU disposition after successful ED stabilization.

OSCARinED links trigger recognition and framework activation to documented treatment targets, structured reassessment, and disposition planning. The framework aims to support consistent, goal-directed care during prolonged ED stays and to reduce omissions in complex, interruption-prone emergency care environments. It does not replace ABCDE algorithms, sepsis pathways, disease-specific guidelines, or ICU admission processes. Instead, it organizes the post-resuscitation interval until ICU transfer, clinical stabilization, transfer to another high-acuity area, or alternative disposition.

The suffix “inED” emphasizes the intended setting: OSCARinED is a safety bridge for the ED, not a substitute for ICU care. After initial stabilization, the team should define goals of care, communicate a coherent plan, and incorporate patient preferences, advance directives, and ceilings of therapy when clinically appropriate. Each OSCARinED cycle should end with a documented “plan–do–review” statement that can be transferred reliably during handover.

### Domain selection and refinement

Domains were selected if they met four pragmatic criteria: (1) relevance to recurrent safety risks during prolonged ED boarding; (2) applicability to non-trauma critical illness after initial stabilization; (3) feasibility for ED teams using commonly available monitoring, medication, documentation, and reassessment workflows; and (4) suitability for repeated team-based review and handover. Particular emphasis was placed on practice elements that are evidence-informed, omission-prone, time-sensitive, and feasible under ED operational constraints.

The draft domains were iteratively refined by the author group based on emergency medicine, intensive care, anesthesiology, and nursing-relevant feasibility considerations, with emphasis on tasks that are time-sensitive, omission-prone, and suitable for repeated reassessment.

### Oxygenation and ventilation (O)

Securing oxygenation and ventilation is a foundational ED competency. Oxygen may be delivered using nasal cannula or mask (± reservoir), high-flow nasal oxygen (HFNC), non-invasive ventilation (NIV), or invasive ventilation. In spontaneously breathing patients, non-invasive strategies should be considered first when clinically appropriate. NIV is particularly suited for hypoxemic failure (e.g., pulmonary edema) or hypercapnic decompensation (e.g., AE-COPD), with oxygen saturation targets tailored to comorbidity (Table 2) (13).

**Table 2:** Target peripheral oxygen saturation values in selected clinical conditions [according to (13)].

Cause/concomitant diseases	Target SpO_2_ (%)
Myocardial infarction	O_2_ administration if SpO_2_ < 90
Acute exacerbation of chronic obstructive pulmonary disease	88–92
Cardiogenic shock	95–98
Post-resuscitation	94–98
Stroke	>94
Ventilated patients in general	90–94
Sepsis or septic shock	92–96
Carbon monoxide intoxication	FiO_2_ 100% independent of the SpO_2_ value

SpO_2_, oxygen saturation measured by pulse oximetry; FiO_2_, inspiratory oxygen fraction.

Airway protection is mandatory when respiratory failure, upper-airway compromise, or impaired consciousness (Glasgow Coma Scale [GCS] ≤ 8 without an immediately reversible cause) threatens ventilation. When intubation is required, video laryngoscopy is the preferred technique, and capnography is essential for tube confirmation and ongoing monitoring (14, 15).

Because invasive ventilation carries iatrogenic risk, lung-protective settings are pivotal: ~6 mL/kg in ARDS (16), and <8 mL/kg predicted body weight even without ARDS, where benefits include shorter ICU/hospital stay and lower mortality (17). Keep peak airway pressure ≤30 cmH₂O, and titrate FiO₂/PEEP using the ARDS FiO₂–PEEP framework to mitigate atelectasis (Figure 1) (16, 18), with regular arterial blood gases guiding reassessment. Optimize minute ventilation for hypercapnia; adjust FiO₂/PEEP for oxygenation failure (Table 3). Adjuncts such as recruitment maneuvers or bronchoscopy may be considered in selected cases, acknowledging inconsistent outcome data and the need for clear indications (19, 20).

**Table 3:** Suggested initial settings for non-invasive and invasive ventilation.

	Non-invasive ventilation [according to (77)]		Invasive ventilation [according to (78)]	
	In case of hypoxia	For hypercapnia	Hypoxia	COPD or asthma
FiO_2_	100% then reduce quickly	30%	80–100%, then reduce quickly	40–100%, then increase if necessary
PEEP	5 mbar	5 mbar	8 mbar	0–5 mbar
Breathing rate	–	–	12–20	6–8
Tidal volume	–	–	6 ml/kg ibw	6 ml/kg ibw
I:E	–	–	1:2	1:3–1:5
Psupp	5 mbar above PEEP	8 mbar above PEEP	–	–

mbar, millibar; PEEP, positive end-expiratory pressure; kg ibw, kilogram ideal body weight; Psupp, support pressure; I:E, ratio of inspiratory to expiratory time.

Practice implication: For OSCARinED implementation, every patient receiving oxygen therapy, NIV, HFNC, or invasive ventilation should have documented oxygenation and ventilation targets, including SpO₂ range, ventilatory mode, escalation criteria, and timing of the next blood gas analysis.

### Sedation and analgesia (S)

Pain is ubiquitous in critical illness and is often intensified by ED procedures. In spontaneously breathing patients, quantify pain with the Numerical Rating Scale (NRS) and target NRS < 4 (21); when opioids are required (e.g., morphine), pair them with a non-opioid adjunct such as paracetamol (Table 4).

**Table 4:** Overview of common analgesics, sedatives, catecholamines and delirium medications.

	Dosage	Side effects (selection, list incomplete)	Special features
Morphine	5–10 mg i.v. 10–30 mg i.m./s.c.	Decrease in appetite, mood changes, euphoria, dysphoria, headache, dizziness, constipation, nausea/vomiting, dyspepsia, miosis, sweating, urticaria, pruritus, urinary retention, respiratory depression	Metabolization into active substances such as morphine-6-glucuronide Antidote: Naloxone
Sufentanil	Initial: 0.15–0.7 μg/kg bw i.v. Maintenance: 0.1–0.25 μg/kg bw/h	Respiratory depression, muscle rigidity, hypotension especially with hypovolemia, bradycardia	Antidote: Naloxone
Remifentanil	initial: Maintenance as direct as possible Maintenance: 1.5–18 μg/kg bw/h = 0.025–0.3 μg/kg/min	Respiratory depression, muscle rigidity, hypotension especially with hypovolemia, bradycardia	Degradation: nonspecific tissue and blood- esterases, thus degradation independent of liver and kidneys Antidote: Naloxone
Propofol	Initial: (1–)1.5–2.5 mg/kg bw i.v. Maintenance: up to 4 mg/kg bw/h i.v.	Respiratory depression to apnea, drop in blood pressure (negative inotropy, reduced peripheral resistance) especially with hypovolemia, agitation phenomena, local injection pain, histamine release, propofol infusion syndrome	Low bronchodilator effect, favorable in traumatic brain injury and increased ICP. If sedation remains insufficient, consider adding or adjusting an analgesic or sedative co-agent rather than exceeding this dose. No antidote available
Midazolam	Initial: 0.15–0.2 mg/kg bw i.v. Maintenance: 0.03–0.2 mg/kg bw/h i.v. Onset of action: 60–90 s Half-life: 1–4 h	Paradoxical excitement Caution: Combination with alcohol (increased alcohol effect), respiratory insufficiency in combination with opioids	Caution: Ampules in different concentrations (5 mg/5 mL and 15 mg/5 ml) Antidote: Flumazenil
Haloperidol	1–10 mg i.m.	Agitation, insomnia, extrapyramidal disorder, hyperkinesia, headache, psychotic disorder, depression, hypokinesia, dizziness, somnolence, tremor, visual disturbances, hypotension, vomiting, nausea, constipation, hypersalivation, dry mouth, urinary retention, rash	No intravenous administration (risk of long QT syndrome)
Clonidine	0.15–0.6 mg i.v., s.c. and i.m. maximum daily dose	Sleep disorders, depressive moods, fatigue, tiredness, headaches, orthostatic dysregulation such as blackness before the eyes, dizziness, tendency to collapse in an upright position and when changing position from lying to standing, dry mouth, constipation, nausea, vomiting, bradycardia	Rapid intravenous administration may lead to a temporary increase in blood pressure; intravenous administration via short infusion;
Dexmedeto-midine	Initially 0.7 μg/kg bw/h, then between 0.2–1.4 μg/kg bw/h variable depending on sedation	Hyper−/hypoglycemia, agitation, bradycardia, myocardial ischemia or infarction, hyper−/hypotension, respiratory depression, nausea, vomiting, dry mouth, withdrawal syndrome, hyperthermia	Administration via syringe pumpe; increased mortality with use > 65 years
Melatonin	2 mg before bedtime	Headache, nasopharyngitis, back pain, arthralgia, hyperbilirubinemia, itching, glucosuria, proteinuria, weight gain, increase in transaminases	
Norepinephrine	PUSH-Dose-Pressor: 10 μg/mL: 1 mg per 100 mL NaCl 0.9%; ml-wise via pVC or cVC Low concentration for pVC: 20 μg/mL: 1 mg to 50 mL NaCl 0.9% via perfusor; run rate depending on the circulatory situation Highly concentrated for cVC: 100 μg/mL: 5 mg to 50 mL NaCl 0.9% via perfusor: rate depending on the circulatory situation Titrate carefully	Hypertension, headache, bradycardia, hyperglycemia, metabolic acidosis	Better responsiveness with balanced volume status and pH
Dobutamine	Usually ready prepared with 5 mg/ml: run rates generally up to 10 mL/h Titrate carefully	Increase but also decrease in blood pressure possible, tachycardia, ventricular arrhythmia, bronchospasm, inhibition of platelet aggregation, nausea, urge to urinate, chest pain	
Epinephrine	In the context of anaphylaxis: 0.5 mg pure i.m.; repeated administration possible In the context of resuscitation: 1 mg i.v. every 3–5 min Via cVC: 5 mg to 50 mL NaCl 0.9%: Run rate depending on response Titrate carefully	Tachycardia, cardiac arrhythmia, Increase in lactate level, metabolic acidosis, hyperglycemia, mydriasis, increase in myocardial oxygen consumption	Better responsiveness with balanced volume status and pH

mg, milligram; μg, microgram; ml, milliliter; i.v., intravenous; i.m., intramuscular; pVC, peripheral venous catheter; cVC, central venous catheter; NaCl, sodium chloride (here, solution); s, second; h, hour; kg bw, kilogram body weight.

For invasive ventilation, emergency anesthesia followed by analgosedation is often required (Table 4); adequate analgesia improves ventilator tolerance, may reduce sedative needs, and can stabilize hemodynamics. While ICU practice favors light sedation (RASS 0/−1) (22, 23), ED procedures may justify short, clearly time-limited deep sedation (RASS −5) during early ECC.

Practice implication: Each reassessment should include a documented pain score or sedation target, explicit justification for deep sedation, and a plan for de-escalation once procedures, transport, or severe ventilator dyssynchrony have resolved.

### Catecholamines, fluid therapy and catheter management (C)

Circulatory failure (“C-problem,” shock) is a frequent, high-mortality emergency in critical illness (24–26), and demands close hemodynamic surveillance with tailored fluids and/or vasopressors.

Vasopressors: Norepinephrine can be rapidly titrated—briefly as push-dose boluses and then via syringe pump (Table 4). Low-dose peripheral infusion is acceptable as a bridge; central venous catheter (CVC) placement should *not* delay vasopressor initiation (27). Optimize catecholamine efficacy by correcting hypovolemia and acid–base derangements.

Fluids: Hypotension is not synonymous with fluid need. Identify “responders” using dynamic assessment: inferior vena cava (IVC) diameter/variability (IVC < 1 cm suggests tolerance; >2.2 cm argues against hypovolemia) (28–30); left ventricular outflow tract velocity-time integral (LVOT-VTI) as a surrogate for cardiac output (<18 cm suggests low output) (31, 32); reassess after ~500 mL balanced crystalloid fluid responsiveness is suggested by an increase in cardiac output (CO) > 15% (33). Alternatives include passive leg raise (~300 mL preload challenge) (34) or pulse-pressure variation >13% in suitable ventilated patients (35) (formula: $\mathrm{\Delta Pp}(\%)=100\frac{\text{PPmax}-\text{PPmin}}{\frac{\left(\text{PPmax}+\text{PPmin}\right)}{2}}$ ). Central venous pressure (CVP) is a poor standalone guide (36, 37). Follow international guidance for blood products (38–40); track input/output to maintain perfusion and avoid overload, targeting urine output >0.5–1 mL/kg/h. In septic shock with large-volume resuscitation, albumin may be considered; gelatine is discouraged (41).

Catheters: Central and arterial lines consume time and carry risk; place them strategically. With reliable peripheral access, deferred CVC after initial stabilization may be reasonable (42). CVC are indicated for agents ill-suited to peripheral delivery (e.g., hyperosmolar solutions) and should be sited (jugular/subclavian/femoral) to fit context (43). Continuous invasive arterial pressure monitoring is appropriate in instability and for repeated blood gases; choose radial/brachial/femoral access pragmatically, using ultrasound guidance to reduce complications (44, 45).

Practice implication: For patients receiving fluids or vasopressors, OSCARinED should prompt documentation of the current perfusion target, vasopressor route, fluid responsiveness assessment, urine output, and whether central venous or arterial access is required immediately or can be deferred.

### Anti-infective therapy (A)

In the ED, infection, sepsis, and septic shock are common drivers of critical illness (46). Standardized “sepsis bundles” operationalize early diagnosis and therapy (47). The Surviving Sepsis Campaign condenses this into a 1-h bundle: repeat lactate, obtain blood cultures, administer broad-spectrum antibiotics according to illness severity and local guidance, give 30 mL/kg crystalloid for hypotension or lactate >4 mmol/L, and start norepinephrine to achieve MAP ≥65 mmHg. Recent German guidance recommends antibiotic administration within 3 h for sepsis and within 1 h for septic shock (41).

Empiric therapy should be paired with a structured search for the infectious focus (e.g., LUCCAASS), while ensuring cultures are obtained *without delaying* treatment (48). Antibiotic choice should reflect prior microbiology (resistograms), recent healthcare exposure, and patient-specific risk; to prevent missed doses, early scheduling of subsequent administrations (next 2–3 days) and a dedicated sepsis SOP are advisable.

Practice implication: For suspected infection or sepsis, OSCARinED should explicitly document cultures, first antibiotic dose, planned redosing times, source-control strategy, lactate reassessment, and responsibility for antimicrobial review after transfer or handover.

### Risk-adapted positioning and prophylaxis (R)

Even during ECC, “small” prophylaxis matters: positioning, ulcer protection, thrombosis prevention, and temperature control.

Positioning: Elevating the head of bed to 30–45° may reduce microaspiration and has been associated with markedly lower pneumonia rates; regular repositioning helps prevent pressure injury (49, 50). Prone positioning improves oxygenation and lowers mortality in moderate–severe ARDS (PaO₂/FiO₂ < 200) when maintained >12 h, but is resource-intensive and risk-prone—thus an ED option only in selected cases after stringent indication review (51–54).

Stress-ulcer prophylaxis: Routine prophylaxis is debated given low bleeding rates (55). Consider it for high-risk constellations (e.g., coagulopathy, liver failure, ventilation >48 h, sepsis/septic shock) (41, 56). PPIs reduce clinically relevant bleeding without clear mortality benefit; comparative data versus H2 receptor antagonists are mixed (PEPTIC vs. meta-analysis) (57–59), and a consistent signal for pneumonia or *C. difficile* has not been proven (57, 60).

Thrombosis prophylaxis: Thromboembolism risk is substantial (≈10–80% depending on ICU indication), supporting early pharmacologic prophylaxis (61–63). Use LMWH when GFR > 30 mL/min/1.73 m^2^; otherwise dose-adjust or use unfractionated heparin (noting potentially reduced subcutaneous absorption under vasopressors/low output) (64, 65).

Temperature management: Avoid hypothermia to limit coagulopathy; after OHCA, maintain 33–36 °C and prevent fever, using physical temperature measures as needed with explicit targets in the care plan (66–68).

Practice implication: At each reassessment, the ED team should confirm patient position, pressure-injury prevention, thrombosis prophylaxis, stress-ulcer prophylaxis when indicated, and temperature targets, because these tasks are easily omitted during ED boarding.

### Electrolytes and blood gas analysis, glucose management and nutrition (E)

Regular arterial blood gases help track glucose and electrolyte derangements in critical illness. Potassium and calcium disturbances require prompt correction, whereas sodium must be normalized gradually. Both hypo- and hyperglycemia correlate with worse outcomes; thus, moderate control is advised (≈7.8–11.1 mmol/L) (69–71). Nutrition should be initiated early—preferably enteral within 48 h of the last meal (72, 73); with parenteral support considered after 3–7 days if enteral/oral feeding is not feasible (74). In the ED’s initial hours, artificial nutrition is usually unnecessary. However, the OSCARinED reassessment loop should document the time of last oral intake, aspiration risk, glycemic control, and whether nutrition planning needs to be handed over to the ICU or receiving ward.

Practice implication: OSCARinED should document the timing of the last blood gas analysis, glucose target, relevant electrolyte abnormalities, time of last oral intake, aspiration risk, and whether nutrition planning must be handed over to the ICU or receiving ward.

### Delirium management and prophylaxis (D)

Delirium is an acute neurocognitive syndrome—*a sign, not a diagnosis*—and mandates rapid identification of reversible triggers (e.g., hypoxia, infection). Routine screening (e.g., Nu-DESC; Table 5) should be embedded into care at predefined intervals and during handovers (75). Prevention should start in the ED: daytime orientation, communication and mobilization (including respiratory therapy), sensory aids and circadian support; at night, protect sleep via noise/light reduction, earplugs/eye masks, and medication review to preserve physiologic sleep (23). Pharmacologic options are summarized in Table 4.

**Table 5:** Nursing Delirium Screening Scale (Nu-DESC) (according to (75)).

Symptoms	Documentation of the points
Disorientation: Manifestation of disorientation to time or place through words or behavior or failure to recognize of the surrounding person	–
Inappropriate behavior: Inappropriate behavior to place and/or person: e.g. pulling on catheters or bandages, attempting to get out of bed when contraindicated, etc.	–
Inappropriate communication: Inappropriate communication: incoherent or no communication at all, nonsensical or incomprehensible verbal utterances	–
Illusion/hallucination: Seeing or hearing non-existent things, distortion of visual impressions	–
Psychomotor retardation: Slowed responsiveness, little or no spontaneous activity/expression, e.g., if the patient is nudged, the reaction is delayed and/or the patient cannot be awakened	–
Application (per category): Not available: 0 points Weakly present: 1 point Pronounced present: 2 points Nu-DESC positive (delirium) if the sum of the points ≥ 2	

Practice implication: Delirium screening should be incorporated into scheduled reassessment and handover, particularly for older patients, mechanically ventilated patients, patients with sepsis, and those receiving sedatives or analgesics.

**Table 6:** OSCARinED implementation components and suggested process indicators.

Component	Practical purpose	Suggested process indicator
Trigger criteria	Identify patients requiring ongoing ED-based ECC	Proportion of eligible patients with OSCARinED activation
Target documentation	Prevent implicit or drifting goals	Documented SpO₂, ventilation, sedation, MAP/perfusion and glucose targets
Reassessment cadence	Maintain continuity during boarding	Reassessment completed within predefined interval
Handover structure	Improve transfer of responsibility	Complete handover across all OSCARinED domains
Anti-infective redosing	Prevent missed doses	Next antibiotic dose documented and administered on time
Delirium screening	Prevent under-recognition	Delirium screen completed at least once per shift
Disposition planning	Avoid passive boarding	Documented escalation, transfer, de-escalation, or ceiling-of-care decision

### Policy and practice implications

OSCARinED has several implications for ED organization, staffing, documentation, and quality governance. First, EDs caring for critically ill non-trauma patients should define local triggers for escalation from initial resuscitation to ongoing ECC. Such triggers may include ongoing invasive or non-invasive ventilatory support, vasopressor use, persistent shock, repeated blood gas abnormalities, anticipated ED boarding, or recurrent intra-ED handovers.

Second, OSCARinED requires clear role allocation. A medical team leader and nursing lead should be responsible for target definition, documented OSCARinED cycle completion, reassessment timing, and escalation or disposition decisions.

Third, OSCARinED should be embedded into local documentation systems to ensure that oxygenation, sedation, perfusion, antimicrobial therapy, prophylaxis, metabolic control, delirium screening, and disposition are not treated as isolated tasks but as a repeated care cycle.

At a governance level, OSCARinED can help EDs define minimum structural and procedural requirements for safe ED-based ECC and identify gaps in monitoring, staffing, documentation, and escalation pathways. Importantly, OSCARinED should not be used to normalize prolonged ED boarding or delay ICU transfer. Instead, it should make the risks of boarding visible, support structured care while transfer is pending, and generate process metrics that can inform local quality improvement and capacity planning.

### Implementation in the ED: triggers, requirements, workflow, and governance

OSCARinED should be activated for non-trauma critically ill adults who meet at least one of the following criteria: (1) ongoing organ support after initial ABCDE stabilization, such as invasive or non-invasive ventilation, high-flow nasal oxygen, vasopressors, or repeated fluid resuscitation; (2) anticipated prolonged ED stay because of ICU boarding, diagnostic or procedural delays, or transfer logistics; (3) recurrent intra-ED handovers; or (4) persistent physiological instability requiring scheduled reassessment and target-based care (Table 6).

Minimum requirements: Reliable ECC delivery requires continuous ECG and pulse oximetry; frequent non-invasive or invasive blood pressure monitoring; capnography when ventilatory support is used; access to arterial or venous blood gas analysis; infusion pumps suitable for vasoactive drugs; and standardized sedation, ventilation, sepsis, and handover workflows. Staffing should include an identified medical team leader and nursing lead, with explicit responsibility for reassessment intervals, documented OSCARinED cycle completion, escalation, and disposition communication.

Workflow and documentation: We propose a simple ED workflow: trigger recognition → OSCARinED framework activation → documented targets → scheduled reassessment → handover and disposition decision. This sequence should be visible in the electronic or paper record and repeated after deterioration, procedures, transport, handover, or major changes in organ support. The one-page bedside checklist implementing the OSCARinED framework (Figure 1) can be used to document targets for each domain (e.g., oxygenation/ventilation, sedation depth, MAP/perfusion, anti-infectives/source control, prophylaxis/positioning, glucose/electrolytes, delirium screening) and to prompt timely review.

Reassessment cadence: For boarded patients, a structured reassessment should occur at predefined intervals (e.g., every 30–60 min in unstable patients and at least every 2–4 h in stable patients), and after any major event (imaging transfer, procedure, handover, change in vasopressor/ventilator settings). The reassessment should explicitly confirm whether targets remain appropriate and whether escalation, de-escalation, or disposition needs revision.

Common pitfalls and safety considerations: Implementation should explicitly address frequent ED pitfalls: unrecognized over-sedation with delayed weaning; iatrogenic hyperoxia; fluid overload in patients with limited fluid tolerance; missed antibiotic redosing during prolonged ED stays; omission of thrombosis or stress-ulcer prophylaxis in high-risk patients; and absent delirium screening. A “stop point” at each reassessment should ensure that goals of care and ceilings of therapy are reviewed and documented when clinically appropriate.

To evaluate implementation, EDs may track pragmatic process metrics such as time-to-antibiotics in suspected sepsis, documentation of oxygenation and sedation targets, MAP/perfusion target attainment, timely antibiotic redosing during prolonged stays, completion of delirium screening at least once per shift, and handover completeness. These measures capture reliability of care delivery and are suitable endpoints for implementation-science designs.

### Reassessment as the core safety mechanism

After initial stabilization, ECC requires a shared treatment plan and repeated reappraisal of targets across all OSCARinED domains. Reassessment should confirm whether previously defined targets remain appropriate, whether new organ dysfunction has emerged, and whether escalation, de-escalation, transfer, or alternative disposition is required.

Any sudden deterioration mandates immediate ABCDE-based reassessment in line with non-trauma resuscitation room algorithms such as (PR_E-)AUD^2^IT (76), followed by renewed OSCARinED goal-setting to match the updated clinical reality (Figure 1). Reassessment should be both time-anchored and event-triggered. It should culminate in a documented decision regarding continuation, escalation, de-escalation, transfer, or alternative disposition. Explicit assignment of responsibility to a medical team leader and nursing lead may improve reliability across repeated ED handovers. Each loop should end with a concise, documented “plan–do–review” statement to anchor the next handover and reduce drift in goals over time.

### Quality indicators and evaluation

OSCARinED should be evaluated as a complex practice and implementation intervention rather than as a single clinical treatment. Potential process indicators include the proportion of eligible patients with documented OSCARinED activation, documentation of oxygenation and ventilation targets, documentation of sedation or analgesia targets, time to vasopressor initiation when indicated, reassessment of perfusion and lactate, time to first antibiotic dose in suspected sepsis or septic shock, documentation of antibiotic redosing during prolonged ED stays, completion of thrombosis or stress-ulcer prophylaxis assessment, completion of delirium screening, and handover completeness.

Potential outcome indicators include adverse events during ED boarding, unplanned intubation, hypotension or hypoxemia episodes, missed medication doses, ICU transfer delays, ED length of stay, ICU length of stay, hospital length of stay, mortality, and patient-centered outcomes. However, because many of these outcomes are influenced by illness severity, staffing, ICU capacity, and hospital-level factors, early OSCARinED evaluations should prioritize feasibility, fidelity, process reliability, and team performance. Suitable study designs include before-after implementation studies, stepped-wedge rollouts, mixed-methods implementation studies, simulation-based evaluations, and cluster-randomized trials where feasible.

## Limitations and future directions

This Policy and Practice Review has several limitations. First, OSCARinED is based on a narrative synthesis of heterogeneous evidence, much of which originates from ICU, peri-ICU, sepsis, ventilation, sedation, and patient-safety literature rather than from ED-specific randomized trials. Second, several OSCARinED domains represent extrapolations of established critical care principles to the ED boarding context. Third, implementation feasibility will vary according to local staffing, monitoring capacity, ED architecture, ICU access, documentation systems, and national regulations. Fourth, OSCARinED has not yet been prospectively validated as an outcome-improving intervention.

Therefore, OSCARinED should be regarded as a pragmatic practice framework and cognitive aid rather than as a proven clinical bundle. Future research should evaluate feasibility, fidelity, process reliability, handover quality, team performance, patient-centered outcomes, and ED operational endpoints. Implementation-science designs, including before–after evaluations, stepped-wedge rollouts, mixed-methods studies, simulation-based testing, and cluster trials, are particularly suitable.

## Conclusion

Critically ill non-trauma patients who remain in the ED after initial stabilization require continuous, coherent, and target-based care until ICU transfer, clinical stabilization, or alternative disposition. OSCARinED provides a pragmatic framework for structuring ED-based ECC across seven recurrent domains and linking them to documented targets, scheduled reassessment, handover, and disposition planning. It is a safety bridge during ED boarding, not a substitute for ICU admission. Its successful implementation depends on local critical care capability, staffing, monitoring, documentation systems, and governance support. OSCARinED can be introduced using trigger criteria, a structured bedside checklist implementing the OSCARinED framework, predefined reassessment intervals, and interprofessional training. Future studies should determine whether this framework improves process reliability, team performance, patient safety, and outcomes among critically ill ED patients.
